# Effect of Water–Solid Mixing Sequence and Crystallization Water of Calcium Sulphate on the Hydration of C_3_A

**DOI:** 10.3390/ma15062297

**Published:** 2022-03-20

**Authors:** Shiju Joseph, Jørgen Skibsted, Özlem Cizer

**Affiliations:** 1 Materials & Construction, KU Leuven, 3001 Leuven, Belgium; ozlem.cizer@kuleuven.be; 2 Department of Chemistry and Interdisciplinary Nanoscience Center (iNANO), Aarhus University, DK-8000 C Aarhus, Denmark; jskib@chem.au.dk

**Keywords:** hydration, kinetics, gypsum, Portland cement

## Abstract

Tricalcium aluminate (Ca_3_Al_2_O_6_: C_3_A) is the most reactive clinker phase in Portland cement. In this study, the effect of the sequence of mixing of C_3_A with gypsum and water on the hydration kinetics and phase assemblage is investigated. Three mixing sequences were employed: (i) Turbula mixing of C_3_A first with gypsum and then with water (T-mix); (ii) Hand mixing of C_3_A with gypsum before mixing with water (H-mix); (iii) Pre-mixing gypsum with water and then with C_3_A (P-mix). The results suggest that there is a considerable difference in the hydration kinetics and hydrate phase assemblage, particularly during the initial stages of hydration. P-mix promotes a higher degree of hydration in the initial minutes and considerably influences the main peak in the calorimetry curve of C_3_A hydration. Effects of calcium sulphate with different amounts of crystallisation water (anhydrite, hemihydrate and gypsum) on C_3_A hydration are also investigated, and it is found that the water of crystallisation does not have a significant impact on the kinetics of reaction or the formed hydrate phase assemblage.

## 1. Introduction

Among the clinker minerals present in Portland cement, tricalcium aluminate (Ca_3_Al_2_O_6_: C_3_A) is the most reactive phase, although it is present in rather low amounts (2–10 wt.%) compared to the principal silicate minerals, alite (C_3_S) and belite (C_2_S) [1]. Despite its low quantity, C_3_A has a crucial impact on the early-age hydration, rheology and setting of Portland cement [1]. When C_3_A reacts with water, the AFm (Al_2_O_3_-Fe_2_O_3_-mono) phase OH-AFm (hydroxy-AFm or h-AFm: $C_{4}{\mathrm{AH}}_{13}$) is initially formed along with $C_{2}{\mathrm{AH}}_{8}$ and both as hexagonal plates [2] (Equation (1)). These hexagonal hydrates are metastable and are later converted to the thermodynamically more stable cubic hydrate of hydrogarnet (C_3_AH_6_) (Equation (2)) [3]. Hydration of C_3_A in the absence of sulphate (Equation (1)) is fast and leads to the flash setting of cement systems.

(1) $$2C_{3}A+21H\to C_{4}{\mathrm{AH}}_{13}+C_{2}{\mathrm{AH}}_{8}$$

(2) $$C_{4}{\mathrm{AH}}_{13}+C_{2}{\mathrm{AH}}_{8}\to 2C_{3}{\mathrm{AH}}_{6}+9H$$

Depending on the relative humidity (RH), C_4_AH_19_ may precipitate instead of C_4_AH_13_ [1,4,5]. Furthermore, in the presence of a carbonate source from the raw materials (e.g., limestone) or atmospheric CO_2_, part of the interlayer OH^–^ ions can be replaced by CO_3_^2^^−^ ions to form the stable hemicarboaluminate (Ca_4_Al(OH)_12_(OH)(CO_3_)_0.5_(H_2_O)_5_) or monocarboaluminate (Ca_4_Al(OH)_12_CO_3_(H_2_O)_5_) phases [6,7,8,9].

Calcium sulphates (CaSO_4_·xH_2_O: C$\bar{S}$H_x_), typically in the form of gypsum (CaSO_4_·2H_2_O: C$\bar{S}$H_2_), are inter-ground with the cement clinkers to avoid flash setting [10,11]. High temperatures in the ball mill upon grinding may result in the decomposition of gypsum into hemihydrate (C$\bar{S}$H_0.5_) or anhydrite (C$\bar{S}$). Thermodynamically, the hydration of C_3_A in the presence of calcium sulphate leads to the formation of the AFt (Al_2_O_3_-Fe_2_O_3_-tri) phase, ettringite (C_6_A${\bar{S}}_{3}$H_32_) (Equation (3)) [12,13,14]. However, previous studies have also reported the presence of h-AFm during this period [15,16,17,18,19]. Upon consumption of calcium sulphate, the hydration of C_3_A proceeds by consumption of ettringite forming monosulphate (C_4_A$\bar{S}$H_6+*y*_: m-AFm) (Equation (4)). Depending on the RH, the amount of water (*y*) can vary from 2 to 10 [5].

(3) $$C_{3}A+3{C\bar{S}H}_{2}+26H\to C_{6}{A\bar{S}}_{3}H_{32}$$

(4) $$2C_{3}A+C_{6}{A\bar{S}}_{3}H_{32}+4H\to C_{4}{A\bar{S}H}_{12}$$

The mechanisms, which control the kinetics of these reactions, have been studied extensively in the literature [15,18,20,21,22,23,24,25]. There is a general consciousness that the mechanism of C_3_A hydration is controlled by dissolution [26]. In the presence of gypsum, Ca^2+^-SO_4_^2−^ ion complexes are adsorbed on the surface of C_3_A, hindering its dissolution [20,21]. These ion complexes are desorbed upon the consumption of gypsum, enhancing the reactive surface area of C_3_A [18], which continues to dissolve, depending on the water activity [27].

A previous study by Pourchet et al. [16] reported that the type of calcium sulphate used could lead to differences in reaction kinetics and formed hydration phase assemblage. Their study used two different mixing methods, where they for studies with gypsum initially mixed gypsum with water and then introduced C_3_A later. When they used hemihydrate as the source for calcium sulphate, they dry-mixed it with C_3_A before mixing it with water. Such an approach was necessary as hemihydrate converts into gypsum when mixed with water. Their results showed that when C_3_A is mixed with gypsum, more of the OH-AFm phase is formed in the early hours than when mixed with hemihydrate. They also noted that the appearance of the main peak of C_3_A hydration in isothermal calorimetry is also affected by the type of calcium sulphate used. However, it is not clear whether these differences are solely a result of the source of calcium sulphate or if there are further dependencies of the mixing procedure. Most recently, Neto et al. [28] have reported that they did not find any major difference in the hydration of the cubic polymorph C_3_A with gypsum or hemihydrate.

In the literature, different mixing procedures have been used for C_3_A hydration, but none of the studies has made a systematic comparison of the mixing procedures to explore their impacts on hydration. This work investigates the effect of different mixing procedures and different calcium sulphate sources (amounts of crystallisation water) on the hydration kinetics and phase assemblage of C_3_A hydration.

## 2. Materials and Methods

Synthetic C_3_A with three different fineness, fine (Af), medium (Am) and coarse (Ac), were obtained from SARL minerals, France. The particle diameters, d10, d50 and d90, determined from laser diffraction, is reported in Table 1. XRD characterisation revealed the cubic polymorph of C_3_A [29] for all samples with minor impurities of mayenite (C_12_A_7_) and portlandite (CH). Analytical grade gypsum, anhydrite and hemihydrate were purchased from Merck (Boston, MA, USA).

Three different mixing protocols were employed in this work. (*i*) Turbula mix (T-mix) —C_3_A and calcium sulphate were weighed at appropriate mass ratios and mixed using a Turbula shaker-mixer for 24 h followed by the addition of water; (*ii*) Hand mix (H-mix) —C_3_A and gypsum was dry mixed for one minute using a spatula before mixing with water; (*iii*) Premix (P-mix)—the appropriate amount of gypsum was mixed with water and C_3_A was introduced afterwards. The pastes were prepared with deionised water at a water to binder (w/b) ratio = 1.0 (unless otherwise stated), in a temperature-controlled room at 20 ± 1 °C.

The heat release was measured using an 8-channel TAM Air isothermal calorimeter (TA Instruments, DE, USA) Approximately 4–5 g paste was used for each measurement. The time from mixing to placing the sample holder in the calorimeter took around 10 min, and the initial 30 min of measurement were omitted for measurements of cumulative heat, as this period is more prone to error.

For ex-situ measurements, pastes were prepared with a 5.0 g binder (10.0 g paste) transferred to 1.5 mL micro-centrifuge tubes, which were sealed with parafilm. Hydration was stopped using a freeze drier (Martin Christ, Germany) (2 h, 0.03 mbar pressure) after crushing the pastes in an agate mortar. Thermogravimetric analysis (TGA) was carried out on a NETZSCH STA 409 PC instrument (Germany) for roughly 25–40 mg sample at a heating rate of 10 °C/min and under N_2_ flow.

X-ray diffraction (XRD) measurements were carried out on a Bruker D2 phaser (CuKα, 0.02 step size, 0.3 s/step) from 5° to 55° 2θ on finely ground samples. Quantification at early ages was performed using the Topas (academic) software and the external standard method [30,31] with corundum as the external standard.

Single-pulse ^27^Al magic angle spinning (MAS) NMR spectra were carried out on select samples at a 600 MHz (14.09 T) Varian Direct-Drive VNMR-600 spectrometer spectrometer. A home-built CP/MAS probe for 4 mm o.d. rotors was used along with high-power ^1^H decoupling and a spinning speed of 13.0 kHz. The VNMRJ spectrometer software was used for spectral integration of the intensities for the ^27^Al central transition (*m* = ½ ↔ *m* = −½) and satellite transitions (±1/2 ↔ ±3/2 and ±3/2 ↔ ±5/2). The ^27^Al centerband for anhydrous C_3_A covers the spectral range from 80–20 ppm [32,33]. The hydration products, including octahedrally coordinated Al resonate between roughly 5–15 ppm and includes ettringite (13.08 and 13.51 ppm) [34], C_3_AH_6_ (12.4 ppm) [33,35] and the AFm phases (10.2 ppm for h-AFm and 11.8 ppm for m-AFm) [33]. The spectral region for these phases were deconvoluted using the *dmfit* software [36].

## 3. Results and Discussion

### 3.1. Effect of Mixing on the Hydration of C_3_A-Gypsum Systems

**Figure 1:**
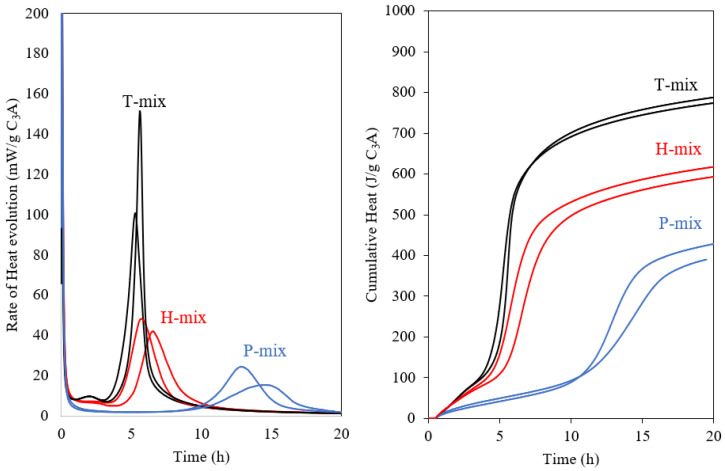
Effect of mixing (Turbula mix–T-mix, hand mix–H-mix and premix–P-mix) on the rate of heat release (**left**) and cumulative heat release (**right**) from isothermal calorimetry for the Af sample with 20 wt.% gypsum and a w/b = 1.0 ratio. Two measurements (multiple lines) were carried out for each sample.

Figure 1 shows the rate of heat release and cumulative heat measured by isothermal calorimetry for the fine fraction of C_3_A (Af) when subjected to the different mixing protocols. The gypsum content (20 wt.% replacement of C_3_A) and the w/b = 1.0 ratio was the same for all mixes. Considerable differences in rate and cumulative heat evolution are observed for the Af samples (Figure 1). The T-mix shows a higher intensity of the main hydration peak for C_3_A and this peak occur earlier as compared to the H- and P-mixes. Moreover, the cumulative heat is also highest for the T-mix. For the H-mix, the occurrence of the main peak is slightly delayed compared to the T-mix and has a much lower intensity. The main peak of the P-mix is found to be even further delayed and broadened. The cumulative heat release measured till 20 h for both the H- and P-mixes were significantly lower than for the T-mix.

**Figure 2:**
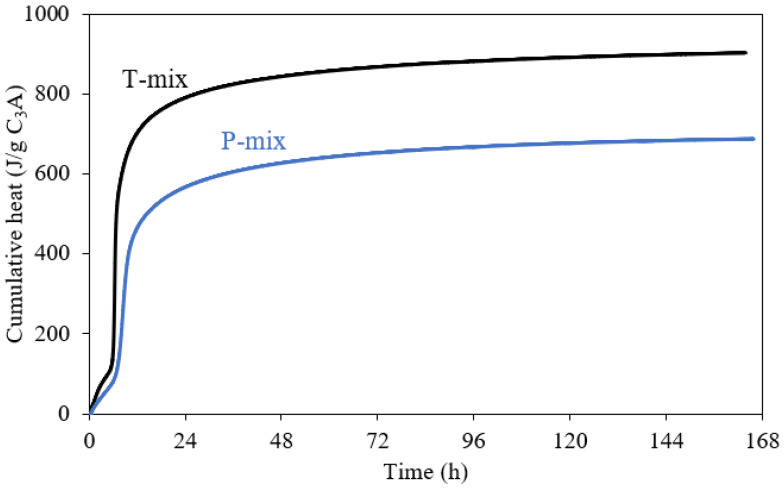
Effect of mixing (Turbula mix–T-mix, and premix–P-mix) on the cumulative heat release measured up to 7 days of hydration from isothermal calorimetry for the Af sample with 20 wt.% gypsum and the ratio w/b = 1.0.

The calorimetry measurements were repeated for the T- and P-mixes to determine the cumulative heat released up to 7 days of hydration (Figure 2). These data show that there is a significant difference in the overall heat released after 7 days, which can reflect a lower degree of reaction for the P-mix or an increased heat release in the early minutes of hydration which is not captured (it is noticed that pastes prepared by the P-mix procedure were much warmer compared to the T-mix).

**Figure 3:**
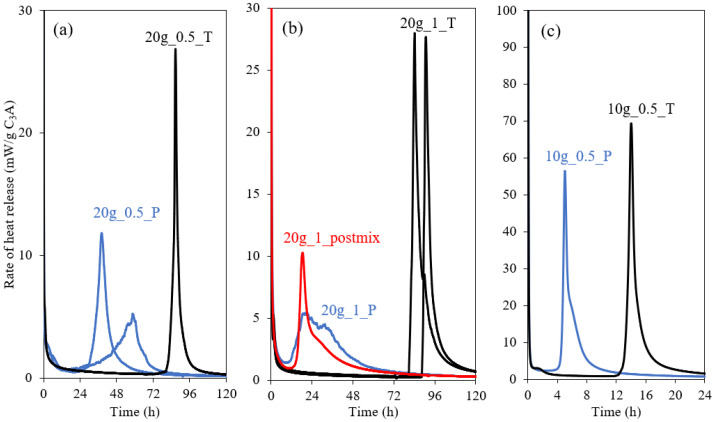
Effect of mixing on the rate of heat release on coarse C_3_A (Ac). (**a**) The effect for the P- and T-mixes with 20 wt.% gypsum and w/b = 0.5; (**b**) the effect for the P-mix, the post-mixing (C_3_A is mixed with water, and gypsum is added after 3 min) and the T-mix with 20 wt.% gypsum and w/b = 1.0; (**c**) the effect for the P- and T-mixes with 10 wt.% gypsum and w/b = 0.5.

The effect of mixing (T- and P-mixes) was further studied on the coarser C_3_A (Ac), with different w/b ratios (0.5 and 1.0) and different gypsum contents (10 and 20 wt.%), as shown in Figure 3. The main peak of C_3_A hydration occurs earlier for the P-mix as compared to the T-mix for all the pastes. This is contrary to the results obtained from Figure 1 and Figure 2 for the fine C_3_A, where the main peak occurs later for P-mix. Still, the main peak for the P-mix appears to be broader and at a lower intensity as compared to the T-mix for both the Ac and Af samples. The main peak occurs also earlier for the sample with w/b = 1.0 relative to w/b = 0.5. In addition, Figure 3b shows the effect of post-mixing (where C_3_A is mixed with water, and gypsum is introduced after 3 min of hydration and then thoroughly mixed), and it is seen that the main peak is similar to that observed for the P-mix.

**Figure 4:**
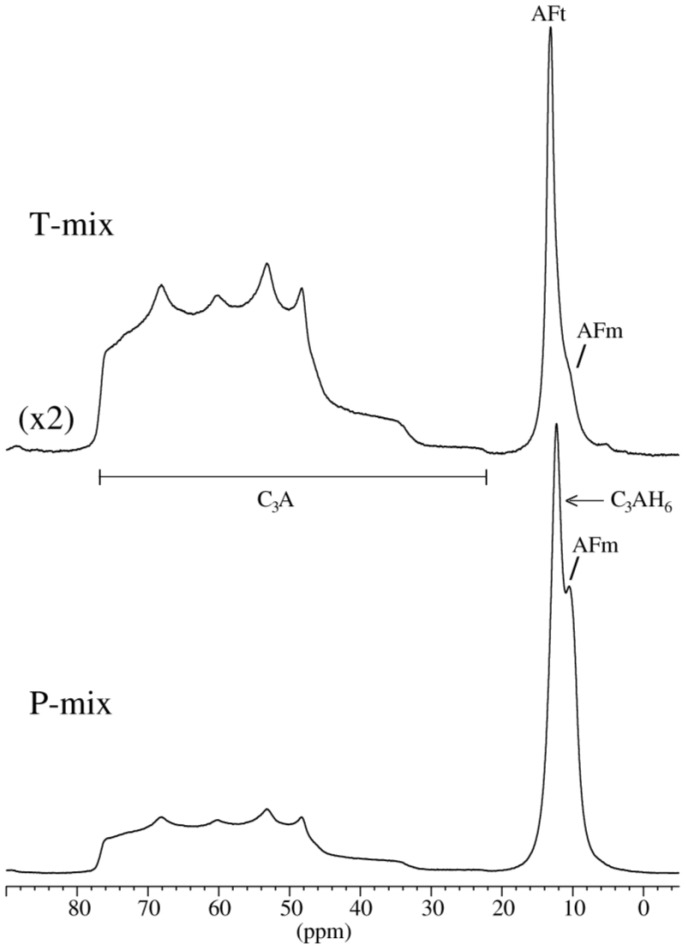
^27^Al MAS NMR spectra recorded at 14.09 T with a spinning speed of 13.0 kHz and illustrating the central-transition region for the P- and T-mixes of the fine C_3_A (Af) hydrated for 3 h. The vertical scale for the spectrum of the T-mix is expanded by a factor 2 relative to the P-mix.

The degree of hydration (DoH) for C_3_A in the different pastes is determined by ^27^Al MAS NMR (Table 2), and two illustrative spectra are shown in Figure 4 for the P- and T-mixes of the fine C_3_A (Af) hydrated for 3 h. It is clearly seen that the degree of hydration at an early age and before the occurrence of the main peak of hydration in the calorimetry curves is considerably higher for the P-mix samples as compared to T-mix pastes. This effect is more pronounced for the fine C_3_A pastes (Af) than for the coarse Ac pastes. On the other hand, the degree of hydration after 7 days is very similar for P- and T-mix samples for both Ac and Af. Comparison of the degrees of hydration with the cumulative heat releases from calorimetry (Table 2) reveals that the differences in heat release from the P- and T-mix pastes result from heat release during the initial minutes after mixing.

**Figure 5:**
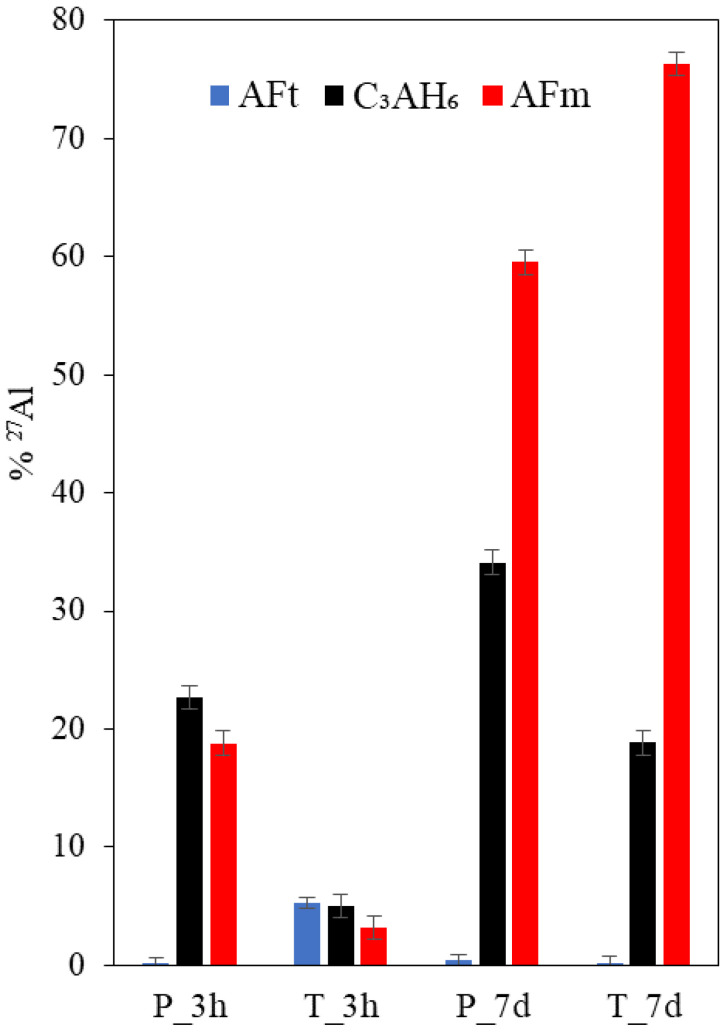
Fraction of ^27^Al present as ettringite/AFt, hydrogarnet (C_3_AH_6_) and AFm phases at 3 h and 7 days of hydration for the P- and T-mix for Af with 20 wt.% gypsum and w/b = 1.0, as determined from ^27^Al MAS NMR.

The relative ^27^Al NMR intensities for the hydrate phase assemblages are shown in Figure 5 for P- and T-mix pastes produced from the fine C_3_A fraction. The intensities have been determined by deconvolutions of the ^27^Al MAS NMR spectra (see Section 2). The P-mix has a very small amount of ettringite but larger amounts of C_3_AH_6_ and AFm phases at three hours of hydration and before the main peak of hydration in the calorimetry curve. On the other hand, the T-mix has a larger amount of ettringite but lower contents of C_3_AH_6_ and AFm phases as compared to the P-mix and apparent from the ^27^Al NMR spectra in Figure 5. After hydration for 7 days, ettringite is virtually absent for both the P- and T-mix pastes, according to ^27^Al NMR, and the T-mix has a higher amount of AFm and lower C_3_AH_6_ content as compared to the P-mix.

**Figure 6:**
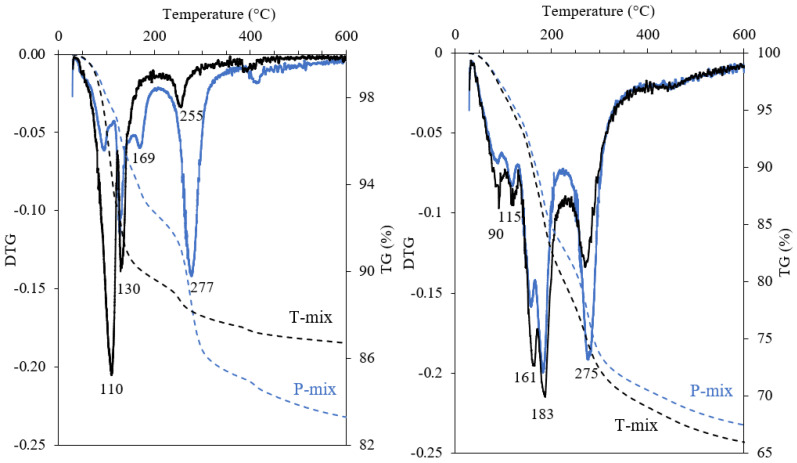
Thermogravimetric analysis for the pastes after hydration for 3 h (**left**) and 7 days (**right**) of the P-mix (blue) and T-mix (black) for the fine fraction of C_3_A (Af) with 20 wt.% gypsum and w/b = 1.0. The lines show the DTG curves and dashed lines the TG mass losses.

The results from thermogravimetric analysis of the Af mixes after 3 h and 7 days of hydration are summarised in Figure 6. After 3 h, a higher mass loss is observed for the P-mix as compared to the T-mix, which corresponds well with the difference in DoH of C_3_A for these pastes from ^27^Al NMR. From the DTG curves, the peak at 110 °C is attributed to ettringite, whereas the peak at 130 °C refers to the release of crystal water of gypsum. Mass loss between 200–400 °C derives from the decomposition of C_3_AH_6_ and AFm phases. By 7 days of hydration, the T-mix has a slightly higher bound water content, which may reflect a higher content of OH-AFm and a lower amount of C_3_AH_6_ relative to the P-mix. This is supported by the higher mass loss for P-mix around 275 °C from C_3_AH_6_ decomposition as compared to the T-mix.

**Figure 7:**
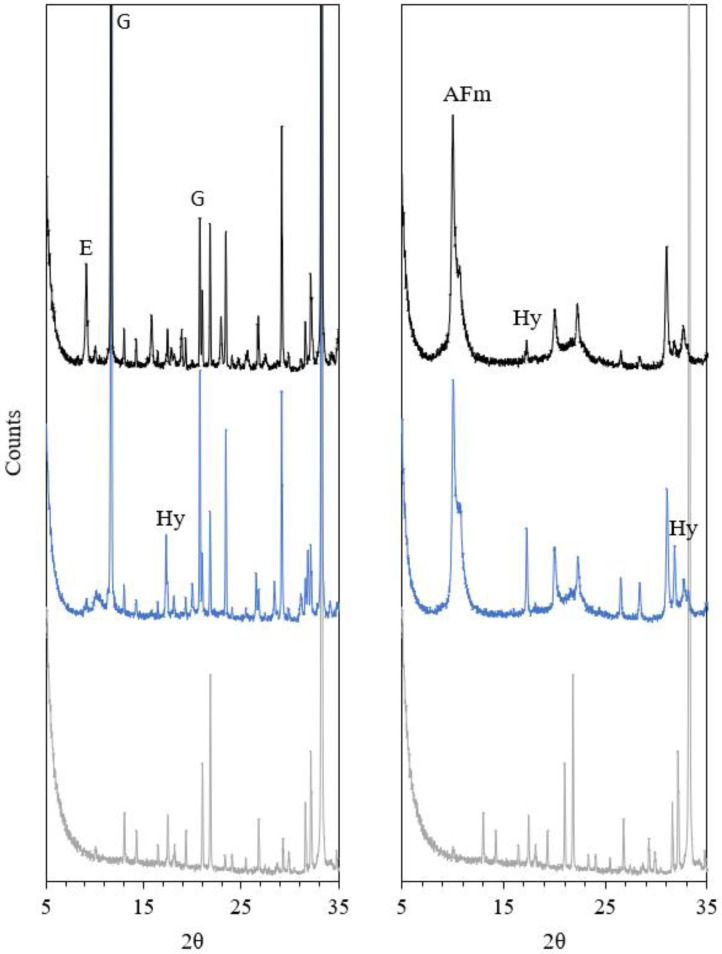
XRD patterns of samples hydrated for 3 h (**left**) and 7 days (**right**) for the P-mix (blue) and T-mix (black) of the Af with 20 wt.% gypsum and w/b = 1.0. The XRD pattern for anhydrous C_3_A is shown as grey. E—ettringite; G—gypsum, Hy—C_3_AH_6_; AFm—monosulphate and hydroxy-AFm.

The XRD patterns of the Af pastes after 3 h and 7 days of hydration are shown in Figure 7. After 3 h of hydration, XRD further confirms the significant amount of ettringite in the T-mix and its nearly absence in the P-mix, which is dominated by C_3_AH_6_ as a hydration product. A higher degree of C_3_A hydration for the P-mix is also clearly visible, in agreement with the ^27^Al NMR results (Table 2). Qualitatively, by 3 h there is a higher consumption of gypsum within the T-mix as compared to the P-mix, although the latter paste has a higher degree of hydration. After 7 days of hydration, mostly AFm phases are present with low crystallinity or short-range order. Hence, it is rather challenging to quantify the phase compositions for these pastes using the Rietveld method. Nevertheless, a higher quantity of C_3_AH_6_ is observed for P-mix, as compared to the T-mix, which corresponds well with the results from ^27^Al NMR and TGA analysis.

Based on the results from calorimetry, TGA, ^27^Al NMR and XRD, it can be concluded that there is a significant effect of the mixing procedure on the hydration kinetics for the C_3_A-gypsum systems. For the premix of gypsum and water (P-mix), gypsum is less efficient in retarding the hydration kinetics of C_3_A in the initial minutes as compared to the paste where C_3_A and gypsum are thoroughly intermixed before hydration (T-mix). This may reflect that gypsum is not homogeneously mixed with C_3_A for the P-mix during the initial minutes of hydration, where water becomes readily in contact with C_3_A. As a result of the low solubility of gypsum, the Ca^2+^ and SO_4_^2−^ ions consumed by the hydration reaction following Equation (3) are not efficiently resupplied by the dissolution of gypsum, thereby favouring hydration of C_3_A with water (Equation (1)) or potentially formation of monosulphate caused by local heterogeneities [37]. The position of the main peak in the calorimetry curves is similar for the post-mix of gypsum and the P-mix (*c.f.* Figure 3), which further validates the hypothesis that the difference originates from an increased reaction of C_3_A with water forming h-AFm. The increase in the C_3_AH_6_ content for the P-mix after 7 days of hydration can be attributed to the prolonged time for stabilisation of the reaction in Equation (2). This corresponds well with a previous study, which showed an increased C_3_AH_6_ content for finer C_3_A particles by 7 days of hydration [18]. This effect will be more pronounced when the fineness of C_3_A is higher, as more C_3_A will react during the initial minutes until gypsum is homogeneously distributed in the paste. This explains the substantial increase in the degree of hydration for the P-mix, as compared to the T-mix, before the main peak of C_3_A hydration in the calorimetry curves with respect to Af and Ac. A higher AFm content for an increased C_3_A fineness was also reported by Minard et al. [15]. Here, the non-linearity between the cumulative heat and initial gypsum content at higher amounts of gypsum, reported by Minard et al. [15], can be attributed to the conversion of h-AFm into m-AFm.

The relative position of the main calorimetry peak of C_3_A hydration is found to be different for the T- and P-mixes of fine (Af) and coarse (Ac) C_3_A pastes. For the Af particles, the peak of the T-mix occurs before the peak of the P-mix, whereas the opposite is found for the Ac particles. This may be explained based on the kinetics for the conversion of h-AFm into m-AFm by the consumption of gypsum and simultaneous hydration reaction of C_3_A with gypsum. While the fineness of C_3_A has hardly an effect on the kinetics of the h-AFm to m-AFm conversion, there is a significant effect of fineness on the C_3_A-gypsum hydration reaction. The high degree of hydration for C_3_A in the P-mix reduces the reactive surface area of C_3_A in Af, thereby increasing the time required for the consumption of gypsum and, consequently, the main hydration peak. On the other hand, in the Ac systems, the hydration of C_3_A-gypsum is slower due to the lower surface area, while the kinetics of the h-AFm to m-AFm conversion ensures faster consumption of gypsum in the P-mix, thereby accelerating the occurrence of the main peak in the calorimetry curves. Since there is clear evidence that the mixing procedure can have a significant influence on the early age hydration and phase assemblage, it would be beneficial to revisit the study of Pourchet et al. [16], as they found differences in kinetics and phase assemblage with different forms of calcium sulfate but on the other hand used different mixing procedures, as described in the previous section. Hence in the next section of the present work, the effect of different amounts of crystallisation water in calcium sulfate on C_3_A hydration is investigated.

### 3.2. Effect of Crystallisation Water of Calcium Sulphate on C_3_A Hydration

**Figure 8:**
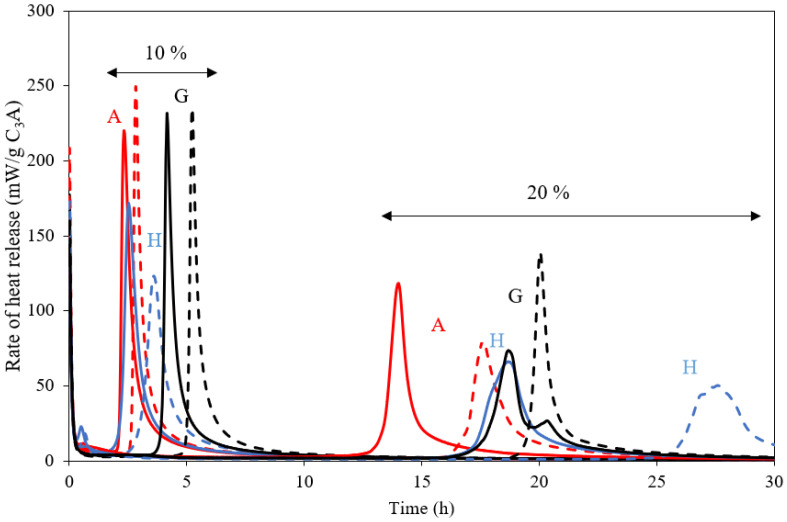
Rate of heat release for C_3_A mixed with anhydrite (A), hemihydrate (H) and gypsum (G) in ratios corresponding to 10 wt.% and 20 wt.% equivalents of gypsum. The solid lines indicate pastes made with 10 g binder and dashed lines 5 g binder.

Figure 8 shows the heat release from isothermal calorimetry for the medium fineness C_3_A (Am) with different forms (C$\bar{S}$H_x_) of calcium sulphate (anhydrite (x = 0), hemihydrate (x = ½) and gypsum (x = 2)). Two equivalent amounts of gypsum (by keeping the Al/S ratio constant) were studied, corresponding to 10 wt.% and 20 wt.% C$\bar{S}$H_2_, and two sets of measurements were collected for pastes made with 5 g and 10 g of anhydrous binder (C_3_A + C$\bar{S}$). The results show consistently that the mass of paste has an effect on the time for the occurrence of the main peak of C_3_A hydration. This can be attributed to an increase in the temperature of the hydrating paste for higher amounts of anhydrous binder. It is well known that hydration of C_3_A is very sensitive to temperature; hence even a marginal increase in the temperature can result in an earlier occurrence of the main peak of hydration. Furthermore, there is heat released due to the additional enthalpy of dissolution for anhydrite and hemihydrate.

For the blends with 10% equivalent gypsum, Figure 8 shows that for the blends with gypsum, the main peak is delayed compared to those of the anhydrite and hemihydrate blends. On the other hand, for the pastes with 20% equivalent gypsum, hemihydrate shows the most delayed occurrence of the main peak, particularly for the pastes made with 5 g binder, whereas the occurrence of the main peak is very similar for the anhydrite and gypsum blends.

**Figure 9:**
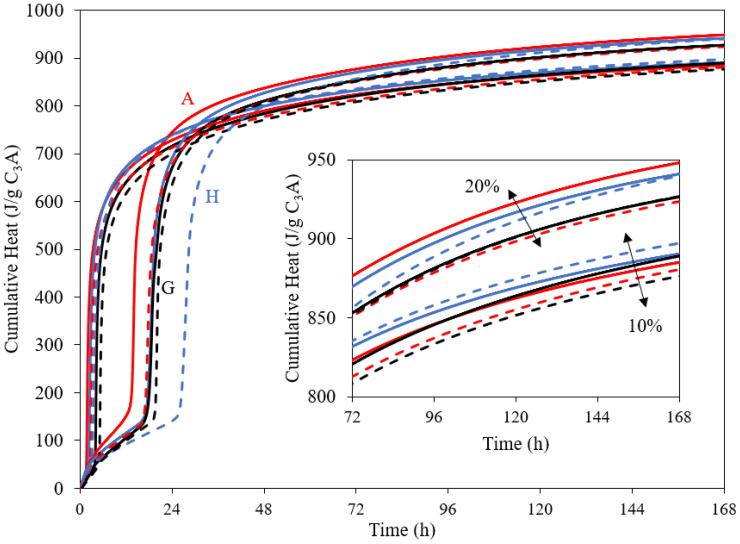
Cumulative heat as a function of hydration time for C_3_A mixed with anhydrite (A, red), hemihydrate (H, blue) and gypsum (G, black) for 10 wt.% and 20 wt.% equivalents of gypsum. The solid line indicates pastes made with 10 g binder and dashed line 5 g binder.

The cumulative heat release from isothermal calorimetry is shown in Figure 9 for the different blends with different C$\bar{S}$H_x_ sources. The pastes with 20% equivalent gypsum are seen to have higher heat releases compared to those with 10%, as expected. However, there is neither a significant difference nor a particular trend for the overall heat release after 7 days of hydration for different levels of crystallisation water of calcium sulphate.

**Figure 10:**
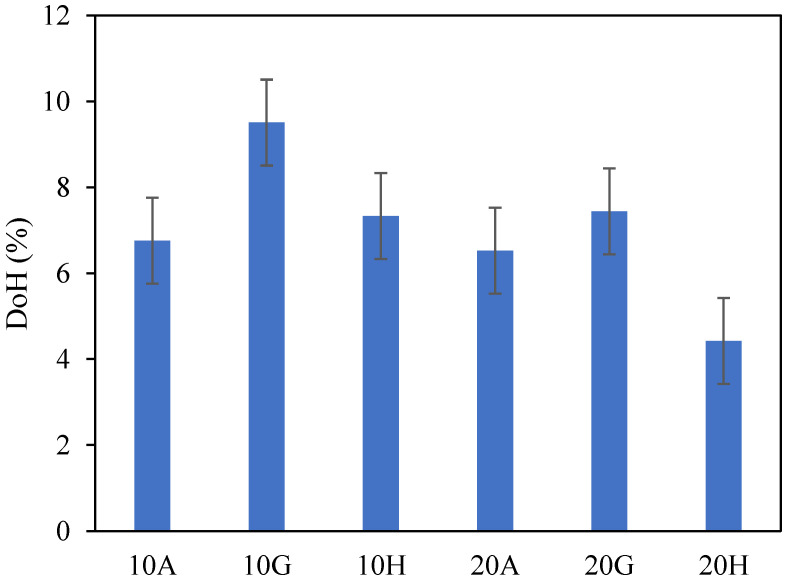
Degrees of hydration for C_3_A after 1 h of hydration for the blends with anhydrite (A), hemihydrate (H) and gypsum (G) determined from XRD/Rietveld analysis; 10 wt.% and 20 wt.% equivalent of gypsum.

Figure 10 shows the degrees of hydration for C_3_A with 10 wt.% and 20 wt.% equivalent gypsum for the blends with anhydrite, hemihydrate and gypsum after 1 h of hydration, and for pastes prepared with 5 g binder to minimise the effect of temperature. The values show that gypsum has a slightly higher degree of hydration after 1 h, and the paste with 20% hemihydrate has the lowest degree of hydration, which is in good agreement with the results from calorimetry.

**Figure 11:**
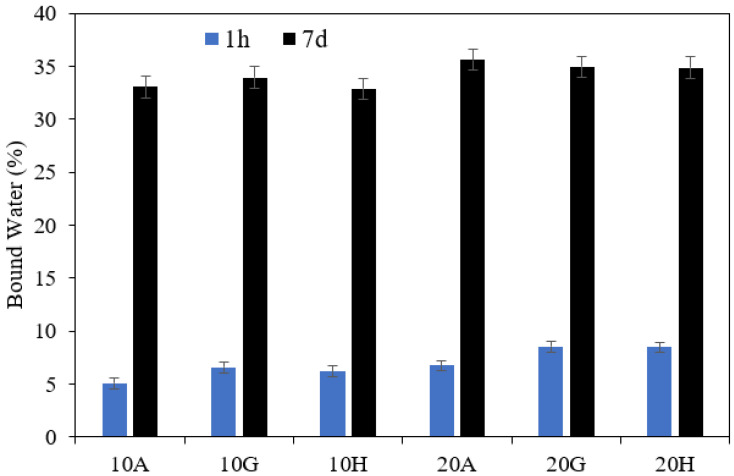
Bound water content determined by TGA for C_3_A mixed with anhydrite (A), hemihydrate (H) and gypsum (G) after 1 h (blue) and 7 days (black) of hydration; 10 wt.% and 20 wt.% equivalent of gypsum.

**Figure 12:**
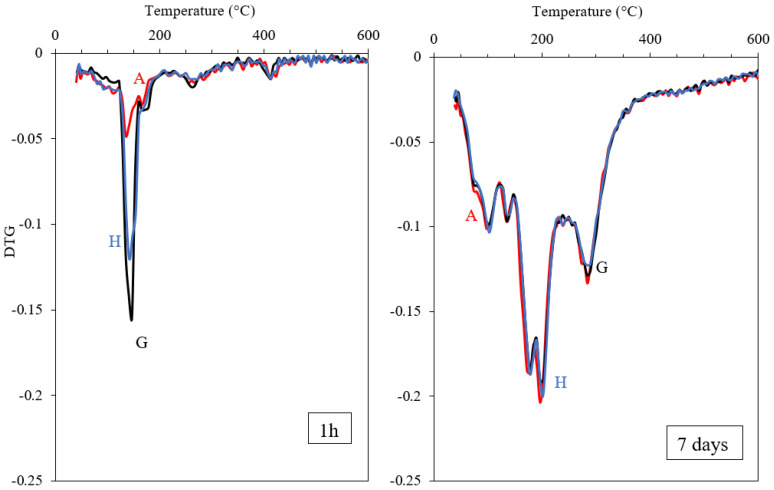
DTG curves for C_3_A mixed with anhydrite (red: A), hemihydrate (blue: H) and gypsum (black: G) after 1 h (**left**) and 7 days (**right**) of hydration; 20 wt.% equivalent of gypsum.

The bound water contents measured after 1 h and 7 days of hydration are shown in Figure 11 for all mixes. This plot shows no significant difference between the calcium sulphate sources after 7 days of hydration; however, the mixes with anhydrite have slightly lower bound water contents after 1 h. The DTG results in Figure 12 indicate that much of the difference between the samples after hydration for 1 h may be attributed to a lower gypsum content. This suggests that a larger part of hemihydrate and a smaller part of anhydrite converts into gypsum during the first hour of hydration. By 7 days, the blends with different calcium sulphate sources show very similar DTG curves, indicating that the hydrate phase assemblage in these samples are very similar.

**Figure 13:**
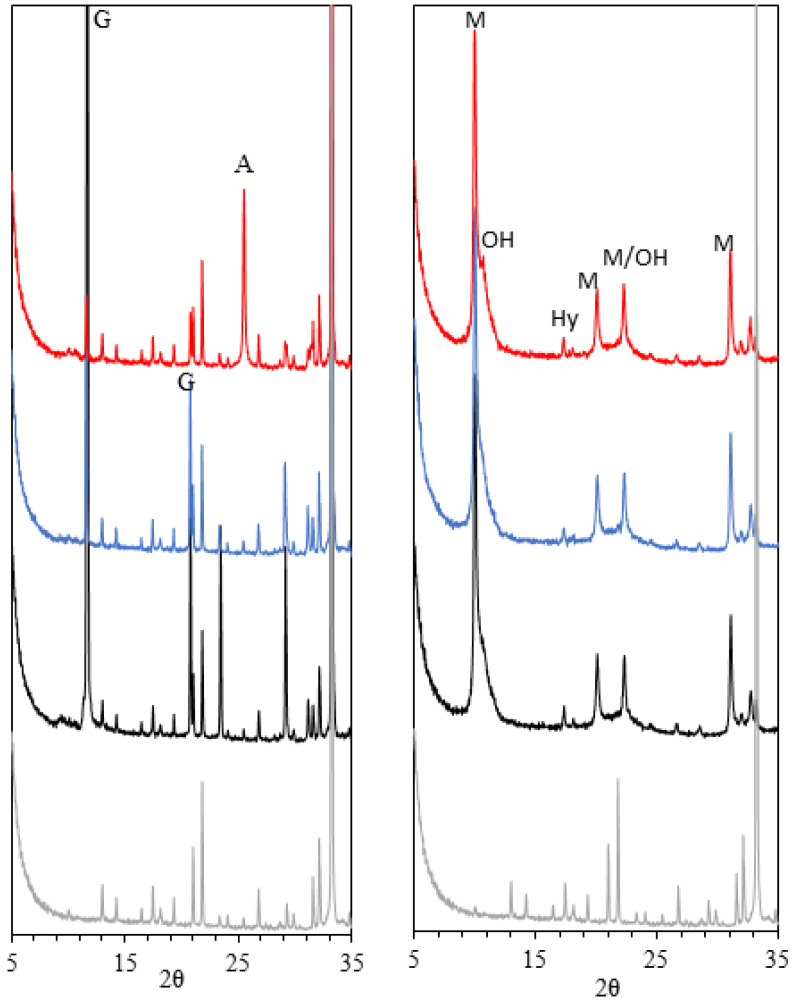
XRD patterns for C_3_A mixed with anhydrite (A, red curve), hemihydrate (H, blue curve) and gypsum (G, black curve) after 1 h (**left**) and 7 days (**right**) of hydration for 20 wt.% equivalent of gypsum. The XRD pattern of anhydrous C_3_A is shown as light grey. M—monosulphate; OH—hydroxy AFm, Hy—C_3_AH_6_.

Figure 13 shows the XRD patterns of all mixes after 1 h and 7 days of hydration and confirm that only a minor part of anhydrite is converted into gypsum after 1 h, contrasting the observations for hemihydrate. After 7 days of hydration, most of the C_3_A has been consumed in all samples and the assemblages of crystalline hydration products are found to be very similar.

Pourchet et al. [16] have reported a higher heat release for C_3_A blended with gypsum during the initial hour of hydration as compared to hemihydrate. Such an effect is not identified in the present work, and the degree of hydration of mixes with gypsum and hemihydrate is found to be very similar. Their study also reported an earlier occurrence of the main peak in the calorimetry curves for blends with hemihydrate and, in particular, for mixes with less than 25% equivalent gypsum. This finding is also in contradiction with our results. Other studies have also reported retardation of the main calorimetric peak for C_3_A in blends with hemihydrate as compared to gypsum, and this was ascribed to the higher solubility of gypsum [38,39]. These differences may be attributed to the different mixing procedures employed by Pourchet et al. [16], as they used the T-mix procedure for hemihydrate and the P-mix approach for systems with gypsum. Their source of C_3_A was also very fine, which contributes to high OH-AFm precipitation in the early minutes and a higher degree of C_3_A hydration. This resulted in a delay of the main peak in the calorimetry curves, as also observed for the Af mixes in the present study. For mixes with >25% equivalent gypsum, Pourchet et al. [16] reported that the main calorimetric peak occurs earlier for gypsum as compared to hemihydrate. This is attributed to the kinetics for the conversion of OH-AFm into m-AFm. At higher percentages of sulphate, there will be retardation of the main peak. In addition, high amounts of OH-AFm produced during the initial minutes of hydration will consume gypsum.

## 4. Conclusions

The effect of different mixing procedures on the hydration kinetics for C_3_A has been investigated for two different degrees of fineness for C_3_A, using a range of complementary analytical tools, i.e., isothermal calorimetry, thermal analysis, X-ray diffraction and ^27^Al NMR spectroscopy. Primarily two different mixing procedures were studied, (*i*) T-mix, where C_3_A and gypsum is mixed in a turbula blender and water is subsequently added to the dry mix and (*ii*) P-mix, where gypsum initially is mixed with water and C_3_A is added afterwards. It is found that a significant difference in the rate of hydration is clearly present for the different mixing procedures. Compared to the T-mix, the P-mix procedure results in a higher degree of hydration during the initial hours of hydration, the main hydration product being OH-AFm. The differences are more pronounced, particularly for C_3_A with higher fineness. These are attributed to a local under-saturation of Ca^2+^ and SO_4_^2−^ ions in the P-mix during the initial minutes of hydration, which fails to hinder the C_3_A dissolution actively. As the effect of mixing significantly influences the kinetics of hydration, the effect of different forms of calcium sulphate (anhydrite, hemihydrate and gypsum) on C_3_A hydration has been revisited. Our findings show that earlier proposed differences in the initial AFm content when mixing C_3_A with hemihydrate and gypsum most likely result from the use of different mixing protocols. Finally, it is found that the C_3_A mixed with different calcium sulfate sources results in very similar degrees of C_3_A reaction and hydrate phase assemblages after prolonged hydration, e.g., 7 days in the present work.

## Figures and Tables

**Table 1:** Fineness of materials determined from laser diffraction.

	d10 (µm)	d50 (µm)	d90 (µm)
C_3_A Fine (Af)	0.3	4.2	26
C_3_A Medium (Am)	0.9	6.7	37
C_3_A Coarse (Ac)	2.6	19	63

**Table 2:** Degrees of hydration (DoH) for C_3_A from ^27^Al MAS NMR and cumulative heat release from isothermal calorimetry.

Mix ID ^a^	Age	C_3_A DoH (%)	Cumulative Heat (J/g C_3_A)
Af_20G_1_P	3 h	41.5	40.2
	7 days	94.1	689
Af_20G_1_T	3 h	13.5	72.7
	7 days	95.4	904
Ac_20G_0.5_P	1 day	12.3	98.2
	7 days	62.6	568
Ac_20G_0.5_T	1 day	7.2	96.8
	7 days	60.7	628

^a^ MixID: C_3_A fineness_% gypsum_w/b ratio_type of mixing.

## Data Availability

Not Applicable.
